# Effectiveness of Credelio^®^ Plus, a novel chewable tablet containing milbemycin oxime and lotilaner for the treatment of larval and immature adult stages of *Toxocara canis* in experimentally infected dogs

**DOI:** 10.1186/s13071-021-04762-x

**Published:** 2021-05-17

**Authors:** Lisa M. Young, Scott Wiseman, Elizabeth Crawley, Dwight D. Bowman, Craig R. Reinemeyer, Daniel E. Snyder

**Affiliations:** 1 Elanco Animal Health Research and Development, 2500 Innovation Way, Greenfield, IN 46140 USA; 2 Elanco Animal Health, Priestley Road Basingstoke, Hants, RG24 9NL UK; 3 College of Veterinary Medicine, Cornell University, Ithaca, NY USA; 4 East Tennessee Clinical Research Inc, Rockwood, TN USA; 5 Daniel E. Snyder, DVM PhD. Consulting, LLC, Indianapolis, IN 46229 USA

**Keywords:** Credelio Plus, Dog, Effectiveness, Lotilaner, Milbemycin oxime, *Toxocara canis*

## Abstract

**Background:**

The ascarid, *Toxocara canis,* is a common and important zoonotic intestinal nematode parasite that infects dogs globally. An effective treatment that kills any pre-patent stages of immature *T. canis* could additionally reduce or eliminate the development of patent infections that can result in clinical disease in infected dogs and would further reduce environmental contamination of eggs. Two randomized, blinded, GCP-compliant, pivotal laboratory dose confirmation studies were conducted to assess the effectiveness and safety of a new novel combination of lotilaner and milbemycin oxime tablets (Credelio Plus) administered orally to dogs that were experimentally infected with immature (L4 or immature adult [L5]) stages of *T. canis.*

**Methods:**

The commercial tablet formulation of Credelio Plus^®^ was administered in a time frame relative to inoculation with infective eggs. This allowed for effectiveness to be assessed against each specific immature stage of *T. canis*. In each study, dogs were randomized and allocated to one of four treatment groups. Each treatment group contained ten dogs that had been experimentally inoculated on Day 0 with infective *T. canis* eggs and then were dosed once on Day 14 or Day 24 using either placebo tablets or Credelio Plus tablets (IP) to provide minimum dosages of 0.75 mg/kg of milbemycin oxime and 20 mg/kg of lotilaner. All dogs were necropsied 5 or 6 days after their respective treatment. At necropsy, all nematodes recovered from the gastrointestinal tract were counted by species and stage.

**Results:**

In both dose confirmation studies using geometric mean worm counts, effectiveness of Credelio Plus was ≥ 98.6% and ≥ 96.8% against L4 larval stage *T. canis* and immature adult [L5] *T. canis* in both studies, respectively.

**Conclusions:**

These studies demonstrated that the Credelio Plus combination tablet administered orally to dogs was highly efficacious against experimental infections with L4 and immature adult [L5] stages of *T. canis.*

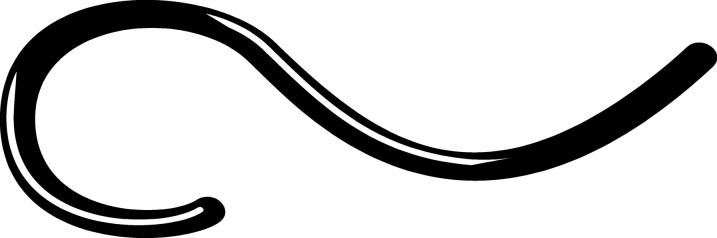

## Background

Dogs are commonly infected and diagnosed with a number of different intestinal nematode species from all parts of the world, including Europe [1–4]. *Toxocara canis* and *Ancylostoma caninum* are considered two of the most important and commonly reported nematode parasites of dogs [5, 6]. It has been documented that dogs infected with *T. canis* play a significant role in the transmission of this potentially zoonotic nematode by excreting eggs directly into the environment where humans and other dogs can be subsequently exposed to infective eggs. In addition, hookworm infections in dogs can also lead to potential veterinary and public health issues [6–8].

There are currently a number of approved endectocidal combination drug products or anthelmintics that have broad-spectrum nematocidal activity. These established drug products can provide the pet owner and veterinarian with the ability to treat dogs that are concurrently infested or infected with multiple parasite types. Milbemycin oxime (MO) has been shown to be effective in combination with other drug substances administered to dogs naturally or experimentally infected with different species of adult intestinal nematodes as demonstrated in both laboratory and field studies [9]. Additionally, it has been shown that a minimum dosage of 0.75 mg/kg of MO will prevent angiostrogylosis by reducing the infection level of immature adult [L5] and adult stages of the French Heartworm *Angiostrongylus vasorum* as well as larval and immature adult stages of *A. caninum* and *T. canis* [10, 11]. The second drug substance in this fixed combination, lotilaner (Credelio^TM^), has demonstrated excellent effectiveness against several ectoparasites (i.e. fleas, ticks, mites) on dogs [12–25].

The objective of both dose confirmation studies as summarized herein was to confirm the dosage of milbemycin oxime in combination with lotilaner (Credelio Plus^®^). Effectiveness was assessed when these combination tablets were orally administered to dogs at approximately the lower end (0.75 to 1.11 mg/kg MO) of the proposed MO unit dose range (0.75 to 1.53 mg/kg MO) that were experimentally infected with immature (L4 and immature adult [L5]) stages of *Toxocara canis.*

## Methods

Use of the lotilaner and MO combination tablets in this study was intended to evaluate the commercial formulation and dose regimen for use in dogs for the intended intestinal nematode label indications. As such, to assess the effectiveness and safety of a combination of lotilaner and MO chewable tablets (IP) administered orally to dogs experimentally inoculated with infective eggs of *T. canis*, these dose confirmation studies were negative-controlled, masked and randomized laboratory studies These studies were conducted as per the principles of GCP as laid down in the VICH guideline GL9, Good Clinical Practice (June 2000); The Efficacy Requirements for Anthelmintics: Overall Guidelines (VICH GL7); The World Association for the Advancement of Veterinary Parasitology (WAAVP) guidelines for evaluating the efficacy of anthelmintics for dog and cats; VICH GLl9 Effectiveness of Anthelmintics and Overall Guidelines: Specific Recommendation for Canines (June 2001) [26–29]. At each study site, personnel involved in recording data and making assessments of intestinal nematode effectiveness and safety were masked to each of the treatment assignments.

### Animals

Purpose-bred parasite-naïve laboratory research Beagles were sourced for each study from a USDA-licensed vendor. The enrolled study dogs were a mix of male/female, were 2 months of age when inoculated on Day 0 with infective *T. canis* eggs, had a body weight range of 3.6–7.5 kg and were acclimated for a minimum of 14 days prior to inoculation with infective eggs of *T. canis*. Dogs were housed singly throughout the study, were fed a balanced commercial dry dog food daily and provided with water *ad libitum*. Each dog was identified individually by an ear tattoo. To ensure dogs were healthy and were eligible to be enrolled into each study, physical examinations were performed during the acclimation period. During the entire study, starting after Day 0, all enrolled dogs were examined at a minimum daily for any adverse events or abnormal clinical signs. Body weight was the primary inclusion criterion so that each selected dog fell into the protocol-specified weight range to ensure that each dog was dosed with a whole tablet(s) to deliver between 0.75 and 1.0 mg/kg of MO, which approximates the lower half of the tablet unit dose range. In both studies, 42 to 44 dogs were orally inoculated with approximately 250 infective *T. canis* eggs once on Day 0. Forty dogs that met the inclusion/exclusion criteria were weighed, randomized and allocated to one of the four treatment groups (10 dogs/group).

### Experimental ascarid inoculations

Prior to inoculation, a fecal sample was taken from each dog to confirm that they were free of any pre-existing patent intestinal nematode infections. Dogs in each study on Day 0 were inoculated orally with 250 infective eggs of *T. canis*. Dogs in each study were orally inoculated with infective eggs using the same method. Calculations needed to determine inoculum volume were recorded. Dogs were checked post-inoculation for vomiting at 1 h ± 30 min. The isolate of *T. canis* used in each study had been recently sourced from a naturally infected dog and had undergone zero passages.

### Treatment

Using a certified scale, each study dog was weighed on the day before their scheduled treatment. Dogs were fed a small portion of a moist canned food prior to their scheduled dosing time point. IP treatments were administered using the commercial Credelio Plus solid oral dosage formulation. These IP treatments were administered in a time frame relative to inoculation with infective *T. canis* eggs so that effectiveness could be assessed primarily against immature larval [L4] or immature adult [L5] stages of *T. canis*. Dogs were treated orally by pilling with the MO + lotilaner combination tablets on Day 14 (to assess efficacy against L4 stages) or on Day 24 (to assess efficacy against immature [L5] adults). A corresponding control group received a vehicle control tablet on Day 14 or Day 24. Treatment day dog observations were performed just after dosing on each scheduled day and at 1 and 2 h as well as 4 and 8 h to confirm that the oral solid dosing formulation had been accepted and that any abnormal health observations were documented.

### Necropsy/worm counts

All dogs treated on Day 14 or 24 were humanely killed as per current animal welfare guidelines and necropsied 5 or 6 days post-treatment in accordance with relevant laboratory procedures. On the day prior to each scheduled necropsy date, food was removed for an overnight fast. During necropsy of each dog, the entire digestive tract was removed. After removal, it was processed in accordance with the relevant standard operating procedures of each laboratory as well as standard parasitological procedures. Due to the small size of immature stages of *T. canis* that were being assessed, the intestinal contents were washed over fine mesh sieves to allow for a complete recovery of all parasites. In addition, each small intestine was soaked separately in warm saline to facilitate the emergence of any immature stages present in the mucosal tissues. The material recovered from each of the soaked intestines was similarly sieved for worm recovery. All worms recovered from each dog were preserved in 10% formalin after which they were counted and microscopically identified to species and stage.

### Variable classification

In the IP and CP groups, the primary variable evaluated was the total count of L4, immature adult [L5] and adult stages of *T. canis* recovered at each post-treatment necropsy day. Each treatment was administered in a time frame relative to inoculation. This allowed for effectiveness to be assessed specifically against L4 or immature adult [L5] stages of *T. canis*. For each of the experimentally induced *T. canis* populations, effectiveness was determined post-treatment for the MO + lotilaner combination by comparing the mean total count with that in the corresponding vehicle control group. Effectiveness of ≥ 90% and a statistically significant difference (*p* < 0.05, two-sided) between each vehicle control group and its corresponding MO + lotilaner-treated group was required to demonstrate effectiveness against the L4 or immature adult [L5] stages of *T. canis*. As outlined in the VICH guidelines, at necropsy a minimum of five specimens of any stage (total of immature L4, immature adult) in each of six control dogs was required in each study to meet the adequacy of infection criteria for each parasite stage of interest.

### Data analysis

The total *T. canis* count in each dog at necropsy in each group was the sum of all recovered worms. For each individual animal's total post-treatment worm count, a logarithmic transformation (ln[count + 1]) was applied to address the skewed nature of these data and also to allow zero counts. Back-transformed geometric means (GM) were calculated as (e^mean^ − 1); the mean was the treatment group arithmetic mean of the log-transformed counts at each given time point. Effectiveness against each stage of *T. canis* was determined using the following formula: [(C − T)/C] × 100, where C was the geometric mean total worm count for the placebo control group and T was the geometric mean total worm count for the IP-treated group. Transformed counts were then analyzed with a general linear model with fixed effect treatment and block as a random effect. To assess the effectiveness of the treatment against immature L4 stages of *T. canis*, statistical contrasts were constructed comparing the L4 control group against L4 in the treated group in each of the studies. In addition, for each study the immature adult control group and the immature adult-treated group were compared to determine the effectiveness of the treatment against the immature adult stage [L5] of *T. canis*. All statistical analyses used the statistical package SAS 9.4 (Cary, NC).

## Results

There were no mortalities and no treatment-related adverse reactions in either laboratory dose confirmation study. Effectiveness results against immature *T. canis* stages were assessed and are summarized in Table 1.

**Table 1:** Effectiveness of a single oral dose of a novel chewable tablet (Credelio Plus) containing milbemycin oxime and lotilaner against induced L4 larval and immature adult [L5] *Toxocara canis* infections in dogs

Study	Stage at time of treatment	Day of inoculation^a^	Day of treatment	Day of necropsy (worm recovery)	Treatment group	*n*	No. of infected dogs	Worm count range	Geometric worm count	Effectiveness compared to vehicle control	
										% efficacy	Effectiveness *P*-value
1	L4 larvae	0	14	19	Vehicle control	10	7	0–20	4.8	–	–
					Credelio Plus^b^	10	0	0–0	0	100	< 0.001
2	L4 larvae	0	14	20	Vehicle control	10	10	2–51	9.9	–	–
					Credelio Plus^b^	10	3	0–3	0.3	96.8	< 0.001
1	Immature adult [L5]	0	24	30	Vehicle control	10	10	4–72	22.1	–	–
					Credelio Plus ^b^	10	4	0–1	.3	98.6	< 0.001
2	Immature adult [L5]	0	24	30	Vehicle control	10	10	5–67	22.3	–	–
					Credelio Plus ^b^	10	4	0–2	0.4	98.3	< 0.001

*n* number of animals per group

^a^Each dog was inoculated with ca. 250 infective *T. canis* eggs

^b^Credelio Plus provided minimum dosages of 0.89 to 1.0 mg/kg of milbemycin oxime in both studies

### L4 *T. canis worm* counts—Study 1

Seven of the ten dogs in the vehicle control group treated on Day 14 had positive *T. canis* worm counts recorded at necropsy. Six of these seven dogs had five or more worms demonstrating an adequate level of infection (Table 1). The GM count for *T. canis* in the vehicle control group was 4.8. *Toxocara canis* were not recovered from any of the ten dogs in the Credelio Plus group thus demonstrating 100% effectiveness against the *T. canis* L4 stage. Post-treatment *T. canis* worm counts were determined to be significantly different (*P*-value < 0.001) between the Credelio Plus group compared to the vehicle control group.

### L4 *T. canis* worm counts—Study 2

All ten dogs in the vehicle control group treated on Day 14 had positive *T. canis* worm counts recorded at necropsy with a range of 2–51 (Table 1). Eight of these ten dogs had five or more worms thus demonstrating an adequate level of infection. The GM *T. canis* count for the vehicle control group was 9.9. *Toxocara canis* were recovered from three of the ten dogs in the Credelio Plus group (range 0–3). The GM *T. canis* count for the Credelio Plus group was 0.3. The effectiveness of the Credelio Plus tablet against immature L4 *T. canis* was clearly demonstrated at necropsy based on worm counts and compared to the vehicle control group. *Toxocara canis* worm counts were determined to be significantly reduced (*P*-value < 0.001) post-treatment with a GM calculated effectiveness of 96.8%.

### Immature adult [L5] *T. canis* worm counts—Study 1

All ten dogs in the vehicle control group (treated on Day 24) had positive *T. canis* counts recorded at necropsy with a range of 4–72 (Table 1). Nine of these ten dogs had five or more worms demonstrating an adequate level of infection. The GM *T. canis* count for the vehicle control group was 22.1. *Toxocara canis* were recovered from four of the ten dogs in the Credelio Plus group (range 0–1). The GM immature adult [L5] *T. canis* count for the Credelio Plus group was 0.3. The effectiveness of the Credelio Plus tablet against immature adult [L5] *T. canis* was clearly demonstrated at necropsy based on worm counts and compared to the vehicle control group. *Toxocara canis* worm counts were significantly (*P*-value < 0.001) reduced post-treatment with a GM calculated effectiveness of 98.6%.

### Immature adult [L5] *T. canis* worm counts—Study 2

All ten dogs in the vehicle control group (treated on Day 24) had positive *T. canis* counts recorded at necropsy with a range of 5–67 (Table 1). All ten dogs had five or more worms thus demonstrating an adequate level of infection. The GM immature adult [L5] *T. canis* count for the vehicle control group was 22.3. *Toxocara canis* were recovered from four of the ten dogs in the Credelio Plus group (range 0–2). The GM *T. canis* count for the Credelio Plus group was 0.4. The effectiveness of the Credelio Plus tablet against immature adult [L5] *T. canis* was clearly demonstrated at necropsy based on worm counts and as compared to the vehicle control group. *Toxocara canis* worm counts were significantly (*P*-value < 0.001) reduced post-treatment with a GM calculated effectiveness of 98.3%.

## Discussion

Both dose confirmation studies clearly demonstrated as summarized in Table 1 that the flavored Credelio Plus chewable tablets administered orally at approximately the lower end of the proposed MO tablet unit dose range (0.75 to 1.53 mg/kg MO) were highly efficacious when treating dogs experimentally infected with immature (L4 and immature adult [L5]) stages of *T. canis* using an actual MO dosage range of 0.89 to 1.0 mg/kg. In these two studies designed to target immature *T. canis* stages, worm counts were reduced by 96.8% to 100% in comparison to a vehicle control group. Larval stages of *T. canis* and *A. caninum* are considered the dose-limiting intestinal nematode species for MO when dosed at 0.5 mg/kg, and thus a minimum MO dosage of 0.75 mg/kg was confirmed to demonstrate adequate and substantial evidence of effectiveness. It is very important that administered doses of MO and other anthelmintics are accurate, based on the approved label dosage (mg/kg) and the dog’s body weight. Even small variations in the administered mg/kg dose level could potentially impact the overall efficacy against different life cycle stages of pathogenic and zoonotic parasites such as *T. canis*. The effectiveness of the combination Credelio Plus (MO + lotilaner) tablet at a minimum dosage level of 0.75 mg/kg MO against both L4 and immature adult stages of *T. canis* was clearly demonstrated in both dose confirmation studies as summarized above.

The ectoparasiticidal component of the IVP, lotilaner, is already registered in the European Union (lotilaner, Credelio^TM^ chewable tablets for dogs) for the treatment of flea (*Ctenocephalides felis* and *C. canis*) and tick (*Rhipicephalus sanguineus*, *Ixodes ricinus*, *Ixodes hexagonus* and *Dermacentor reticulatus*) infestations in dogs and additionally has been shown to be effective against other global tick and mite species [12–25]. To extend the effectiveness of the commercial drug product when dogs have concurrent nematode infections and ectoparasite infestations, lotilaner was combined with MO to form Credelio Plus. Use of a broad-spectrum endectocidal drug product like Credelio Plus on a routine basis can play a significant role in the control of multiple endo- and ectoparasites in dogs. The use of Credelio Plus by pet owners and veterinarians will provide control of larval stages of *T. canis*, adult *T. canis* along with other adult and larval intestinal nematode species on the product label that are commonly reported from dogs globally as well as in Europe [2–4, 6–8, 30–34].

Diagnosis of immature stages of *T. canis* infections can now be based on new ELISA assays using coproantigen detection [35, 36]. These ELISA coproantigen assays are sensitive and specific and have the potential to diagnose *T. canis* infections when standard fecal egg counting flotation methods cannot detect such pre-patent infections. Combination drug products such as Credelio Plus that have demonstrated effectiveness against larval L4 and immature adult stages of *T. canis* can play a role in the overall epidemiology of this zoonotic parasite. Killing these immature stages of *T. canis* prior to the infection becoming patent will reduce environmental contamination. This is important since these eggs can remain infective for many years in the environment thus leading to a continued source of exposure and infection for both dogs and humans [7, 8].

The epidemiology and public health importance of toxocarosis is well documented as a zoonosis of global importance, including in Europe [7, 8, 37, 38]. Human exposure to *T. canis* based on seroprevalance data also has been reported in different regions of the world where the age-adjusted *Toxocara* seroprevalence was 13.9% [39]. Common control measures for *T. canis* and other zoonotic nematode species include regular and frequent anthelmintic treatment of dogs starting at an early age, education and enforcement of laws for the disposal of canine feces, dog legislation and personal hygiene [37]. *Toxocara canis* has been shown to be routinely found in both juvenile and mature dogs [40]; thus, it is important to perform routine monitoring and provide treatment to all age classes of dogs, and this should be a routine practice.

Conducting periodic fecal examinations and treating dogs for *T. canis* is important and should include dogs maintained in the home environment as well as pets that visit places where contact with other dogs or areas that are contaminated by dog feces because these circumstances may support transmission of parasites [2–4, 30–34]. The use of Credelio Plus as a broad-spectrum endectocide that pet owners and veterinarians can use to effectively treat dogs with adult and immature intestinal nematode infections supports the recommendations from ESCCAP and other scientific expert groups to provide regular treatment and control of all intestinal nematodes of dogs and cats [41]. If *T. canis* is a concern based on the age of the dog, past infections or exposure to contaminated areas when dogs are housed outside or have access to the outdoors, under these conditions deworming at least four times a year should be considered. ESCCAP further points out that the routine treatment and prevention of all intestinal parasites depends upon legislation in individual countries, veterinary professionals taking local epidemiological circumstances into account, owner perception and individual risk assessments (e.g. hunting pets, previous lungworm exposure, raw meat diets, etc.). Deworming practices should therefore always be based on the advice of a veterinary professional. The use of Credelio Plus is of particular importance for zoonotic species like *T. canis* as well as zoonotic and pathogenic hookworm species such *A. caninum* that are commonly found in dogs of all ages and throughout the year in prevalence studies conducted in different areas of the world and that include healthy well-cared for dogs in Europe [2–4, 30–34]. There are few data about the impact of re-treatment intervals on parasite burdens and how this might affect environmental contamination on which to base a maximum re-treatment interval. Some data suggest that annual treatments or treatments administered twice a year do not have a significant impact on preventing patent intestinal nematode infections within a population of dogs. Thus, a treatment frequency of at least three to four times per year is a general recommendation that may reduce fecal egg shedding and environmental contamination [41, 42]. Treating dogs with drug products that are labeled to be administered monthly can largely prevent patent infections. These monthly treatment intervals take into account the biology and pre-patent period of these different intestinal nematode parasites. A study that analyzed data from a population of well cared for dogs that were treated with broad-spectrum endectocides on a monthly basis demonstrated that the prevalence of common nematode and cestode endoparasites declined significantly [43]. A minimum dosage of 0.75 mg/kg of MO, as present in Credelio Plus and as confirmed in the studies presented herein, has also been previously documented to effectively treat dogs with L4 larvae and immature adult stages of *A. caninum* and to reduce the level of infection with the lungworm, *Angiostrongylus vasorum*, thus preventing the establishment of patent infections and reducing environmental contamination [10, 11].

MO, alone or in combination with other oral parasiticides, has been used safely for > 20 years for intestinal nematode control and heartworm prevention in dogs [44]. Lotilaner (Credelio) as a standalone product administered as an oral ectoparasiticide has demonstrated safety for dogs under both field use and laboratory conditions [12–25].

## Conclusions

These studies confirmed the effectiveness of a single oral dose of a novel, chewable tablet containing milbemycin oxime and lotilaner (Credelio Plus) against immature stages of *T. canis* infections in dogs. This new combination treatment option of lotilaner plus MO can be used to provide broad-spectrum parasite control for dogs because it offers flea and tick prevention and control, adult and larval intestinal parasite treatment and control, and heartworm and lungworm disease prevention. This will contribute to better owner compliance and simplifies the treatment recommendations from global scientific expert groups and veterinarians to prevent or treat these parasites as well as decrease the transmission of important zoonotic parasites and tick- and flea-transmitted disease agents.

## Data Availability

The dataset summarizing and supporting the conclusions of this article are included within the article. Due to commercial confidentiality of the research, data not included in the manuscript can only be made available to bona fide researchers subject to a fully executed non-disclosure agreement.

## Abbreviations

ESCCAP: European Scientific Counsel Companion Animal Parasites
GM: geometric mean
IP: investigational product
MO: milbemycin oxime
